# Dietary Fat and Risk for Type 2 Diabetes: a Review of Recent Research

**DOI:** 10.1007/s13668-018-0244-z

**Published:** 2018-09-21

**Authors:** Beth H. Rice Bradley

**Affiliations:** Department of Nutrition and Food Sciences, University of Vermont, 109 Carrigan Drive, 255 MLS Carrigan Wing, Burlington, VT 05405 USA

**Keywords:** Dietary fat, Fat, Monounsaturated fat, Polyunsaturated fat, Saturated fat, *Trans*-fat, Fatty acids, Diabetes, Type 2 diabetes

## Abstract

**Purpose of Review:**

It is estimated that over 400 million people worldwide are living with diabetes. Excess adiposity is the strongest risk factor for non-insulin-dependent diabetes, type 2. Lifestyle interventions have demonstrated that diet plays a critical role in preventing the onset of type 2 diabetes. Dietary fat is not only a source of energy and nutrients, but also bioactive fatty acids.

The purpose of this review was to examine data from recent prospective cohort studies and dietary interventions to determine if there are benefits to fat consumption on diabetes risk.

**Recent Findings:**

The consumption of fish and marine *n*-3 fatty acids among Asian populations and regular-fat dairy foods and *trans*-palmitoleic acid (*trans-*16, *n*-7) among Western populations may be associated with reduced risk for type 2 diabetes.

**Summary:**

Whereas some dietary fat may contribute to reduced diabetes risk, lifestyle recommendations to balance calories with physical activity are prudent at this time.

## Introduction

Diabetes mellitus, a group of metabolic disorders characterized by increased blood glucose concentration, increases the risk for morbidity and mortality [1]. It was estimated in 2015 that worldwide, 415 million people, 20–79 years of age, were living with diabetes and 5 million deaths could be attributed to it [1]. The global estimated healthcare costs associated with the disease were 673 billion US dollars [1]. Further, it is estimated that by 2040, the number of people living with diabetes will increase by approximately 55% [1].

The dramatic global increase in the prevalence of diabetes is associated with the global obesity epidemic, particularly because excess adiposity is the strongest risk factor for non-insulin-dependent diabetes, also known as type 2 diabetes [2, 3]. The Diabetes Prevention Program, a lifestyle intervention that promotes weight loss through energy-restriction and physical activity, was shown in a clinical trial to reduce the incidence of type 2 diabetes more effectively than pharmacological intervention, indicating that diet can play a critical role in preventing the onset of type 2 diabetes [4].

Whereas it is recognized that an overall healthy dietary pattern that promotes 30–40% of calories from low-glycemic index carbohydrates is effective at promoting improved glycemic control [5], the effects of dietary fat on diabetes risk are less understood. Dietary fat is not only a source of energy and nutrients, but also bioactive fatty acids that affect cell metabolism [6]. The purpose of this review was to examine the data from recent prospective cohort studies and randomized clinical trials to determine if there are benefits to fat consumption on risk for type 2 diabetes.

## Search Strategy and Selection Criteria

PubMed was searched for original research articles and meta-analyses/systematic reviews conducted in humans over the last 10 years (2008–August 2018). Search terms included “dietary fat,” “monounsaturated fat,” “polyunsaturated fat,” “saturated fat,” and “*trans*-fat” in combination with “diabetes,” “type 2 diabetes,” and “diabetes risk.” Prospective cohort studies and randomized clinical trials that assessed incidence and risk of type 2 diabetes were included in the review. The reference lists of selected articles were also reviewed for relevant literature.

The search yielded mostly articles published on observational findings from prospective cohort studies. Dietary data collected from food frequency questionnaires and fatty acid biomarker data collected from plasma and serum samples indicated that the majority of research pertaining to dietary fat and fatty acids and risk for type 2 diabetes can be categorized by classes of fatty acids, such as *n*-3, saturated, *trans*-, monounsaturated, and polyunsaturated, as well as by their primary food sources, such as fish, meat and dairy, and plant oils. Each of these categories is discussed in this review.

## Associations Between Fish, Marine Sources of *n*-3 Fatty Acids, and Risk for Type 2 Diabetes

A body of prospective research investigating the associations between fish (a predominant source of *n*-3 fatty acids) and *n*-3 fatty acid consumption on risk for type 2 diabetes indicates opposing findings depending on geographical location of study. According to findings from five meta-analyses, whereas fish and marine *n*-3 fatty acid consumption among Asian populations was associated with a decreased risk for type 2 diabetes, fish and marine *n*-3 fatty acid consumption among western Europeans and Americans was associated with increased incidence of the disease [7–9, 10•, 11]. Zhou et al. [11] reported that consuming fish about four times per month and consuming about 0.1 g of marine *n*-3 fatty acids per day increased the risk for type 2 diabetes. These results were based on 13 prospective cohort studies conducted in Western populations. Zheng et al. [10•], however, showed that in pooled analyses from 24 prospective cohorts, that whereas studies conducted in Western populations observed positive associations between fish and marine *n*-3 fatty acid consumption and type 2 diabetes, those studies conducted in Asian populations observed an inverse association. Meta-analyses by Wu et al. [9] and Wallin et al. [8], which utilized many of the same data sets, also reported opposing risks depending on geographical location of study. Muley et al. [7] reported similar findings but indicated that marine *n*-3 fatty acids were associated with reduced risk for type 2 diabetes in Australian cohorts as well as in Asian cohorts. A more recent prospective study from the Australian Longitudinal Study of Women’s Health, conducted in 8370 women, 45–50 years of age, however, indicated that total *n*-3 fatty acids were associated with a 55% increased risk of incident type 2 diabetes in Australian women (Table 1) [17]. These data indicate that fish and marine *n*-3 fatty acid consumption may reduce risk for type 2 diabetes among Asian populations but may be detrimental to Westerners.

**Table 1:** Prospective cohort studies published between 2008 and August 2018 that investigated the associations between dietary fat, fatty acids, and food sources of fat and risk for diabetes

Reference	Region	Study objective	Population	Dietary assessment	Adjustments	Time	Results	Risk
Kaushik (2009) [12]	USA	Investigate the association between dietary long-chain FA and incidence of T2D	195,204 American adults, 24–78 years of age, from three prospective cohorts (Nurses’ Health Study (NHS), NHS 2, and the Health Professionals Follow-Up Study)	Validated FFQ	Smoking, alcohol consumption, physical activity, family history of diabetes, BMI, saturated fat, *trans*-fat, linolenic acid, linoleic acid, caffeine, cereal fiber, glycemic index, calories	14–18 years	Highest quintile vs. lowest quintile of long-chain FA (RR = 1.24; 95% CI 1.09, 1.40) was associated with T2D. Association existed across all quintiles	↑
Brostow et al. (2011) [13]	Singapore	Investigate the associations between total *n*-3 FA, EPA, DHA, non-marine α-linolenic acid, *n*-6 FA, and *n*-6:*n*-3 FA ratio and T2D risk	43,176 Chinese men and women living in Singapore, 45–74 years of age, free from diabetes at baseline, from the Singapore Chinese Health Study	Semi-quantitative FFQ	Fully adjusted model: age, sex, interview year, dialect, hypertensive status, smoking, alcohol frequency, education, BMI, physical activity, hypertension, dietary factors	5.7 years	Highest quintile vs. lowest quintile of total *n*-3 PUFA (HR = 0.78; 95% CI 0.65, 0.94) and non-marine α-linolenic acid (HR = 0.79; 95% CI 0.67, 0.93) were inversely associated with validated self-reported incidence of diabetes	↓
							EPA, DHA, *n*-6 PUFA, and *n*-6:*n*-3 PUFA ratio were not associated with T2D	↔
Djoussé et al. (2011) [14]	USA	Investigate the association between *n*-3 FA and fish consumption and T2D risk	36,328 women, 54.6 year of age on average, free from diabetes at baseline, from the Women’s Health Study	Validated FFQ	Age, BMI, parental history of diabetes, smoking, exercise, alcohol, menopausal status, red meat intake, quintiles of energy intake, linoleic acid, α-linolenic acid, dietary magnesium, *trans* and saturated fats, cereal fiber, glycemic index	12.4 years	Highest quintile vs. lowest quintile of marine *n*-3 FA (HR = 1.44; 95% CI 1.25, 1.65) were positively associated with validated self-reported incidence of diabetes	↑
							Plant-based *n*-3 FA were not associated with incident diabetes	↔
Margolis et al. (2011) [15]	USA	Investigate the association between low-fat dairy consumption and incident T2D	82,076 multiethnic postmenopausal women 50–79 years of age, free from diabetes at baseline, from the Women’s Health Initiative Observational Study	Semi-quantitative FFQ	Age, race/ethnicity, total energy intake, income, education, smoking, alcohol consumption, use of postmenopausal hormone therapy, physical activity, family history of diabetes, BMI, blood pressure, dietary factors	8 years	Highest quintile vs. lowest quintile of low-fat dairy consumption (RR = 0.65; 95% CI 0.44, 0.96) was inversely associated with risk of T2D	↓
							High yogurt consumption (≥ 2×/week) (RR = 0.46; 95% CI 0.31, 0.68) was inversely associated with risk of T2D	↓
							High-fat dairy consumption was not associated with risk of diabetes	↔
Villegas et al. (2011) [16]	China	Investigate the associations between fish, shellfish, and long chain *n*-3 FA and the risk of T2D	51,963 Chinese men and 64,193 Chinese women, 40–74 years of age, free from diabetes at baseline, from the Shanghai Men’s Health Study and the Shanghai Women’s Health Study	In-person interview using a validated FFQ	Age, energy intake, waist-to-hip ratio, BMI, smoking, alcohol consumption, physical activity, income level, educational level, occupation, family history of diabetes, hypertension, and dietary pattern	Approximately 5–10 years	Highest quintile vs. lowest quintile of shellfish (HR = 0.86; 95% CI 0.76, 0.99) and long-chain *n*-3 FA intakes (HR = 0.84; 95% CI 0.74, 0.95) were inversely associated with T2D in women. Inverse associations existed across all quintiles	↓
Alhazmi et al. (2013) [17]	Australia	Investigate the association between macronutrient intake and T2D risk	8370 Australian women, 45–50 years of age, free from diabetes at baseline, from the Australian Longitudinal Study of Women’s Health	Validated FFQ called the Dietary Questionnaire for Epidemiological Studies	Fully adjusted model: lifestyle, sociodemographic factors, other fat types, fiber, energy	6 years	Highest quintile vs. lowest quintile of MUFA (RR = 1.64; 95% CI 1.06, 2.54), total *n*-3 PUFA (RR = 1.55; 95% CI 1.03, 2.32), α-linolenic acid (RR = 1.84; 95% CI 1.25, 2.71), and total *n*-6 PUFA (RR1.60; 95% CI 1.03, 2.48) were positively associated with validated self-reported incidence of T2D	↑
							No associations between total dietary carbohydrate, protein or fat with T2D	↔
Louie et al. (2013) [18]	Australia	Investigate the associations between baseline consumption of dairy products and incidence of metabolic syndrome and T2D	1807 (included in study of metabolic syndrome) and 1824 (included in study of T2D) Australian men and women 49 years of age and older, from the Blue Mountains Eye Study	Validated semi-quantitative FFQ	Fully adjusted model: age, sex, smoking status, physical activity, dietary glycemic load, fiber from vegetables, total energy intake, family history of T2D, systolic blood pressure, BMI, HDL-cholesterol, total cholesterol, triglyceride, calcium	10 years	Highest quartile vs. lowest quartile of regular-fat dairy consumption (OR = 0.41; 95% CI 0.23, 0.71) was inversely associated with risk of metabolic syndrome	↑
							Total dairy consumption was not associated with risk of metabolic syndrome or T2D	↔
Ericson et al. (2015) [19]	Sweden	Investigate the associations between dietary fat and its food sources and T2D risk	26,930 men and women, 45–74 years of age, free from diabetes at baseline, from the Malmö Diet and Cancer Cohort	Interview-based modified diet-history method that combined diet recall, FFQ, and dietary assessment interview	Energy, age, sex, method version, season, leisure time, physical activity, smoking, alcohol intake, education, BMI	14 years	Highest quintile vs. lowest quintile of high-fat dairy consumption (HR = 0.77; 95% CI 0.68, 0.87) was inversely associated with incidence of T2D	↓
							Highest quintile vs. lowest quintile of meat consumption was associated with increased risk for T2D (HR = 1.36; 95% CI 1.20, 1.55)	↑
							Highest quintile vs. lowest quintile of saturated FA with 4–10 carbons (HR = 0.83; 95% CI 0.74, 0.93), lauric acid (12:0) (HR = 0.84; 95% CI 0.75, 0.95, and myristic acid (14:0) (HR = 0.83; 95% CI 0.74, 0.94) were associated with decreased risk for T2D	↓
							Total dietary fat was not associated with T2D	↔
Guasch-Ferré et al. (2015) [20]	USA	Investigate the association between olive oil consumption and incident T2D	59,930 women 37–65 years of age, free from diabetes at baseline, from the Nurses’ Health Study (NHS) I and 85,157 women, 26–45 years of age, free from diabetes at baseline, from the NHS II	Validated FFQ	Ethnicity, ancestry, smoking status, alcohol, physical activity, family history of diabetes, history of hypertension, history of hypercholesterolemia, multivitamin use, postmenopausal status, menopausal hormone use, quintiles of Alternative Healthy Eating Index, total energy intake	22 years	>1 tablespoon of olive oil (>8 g) per day vs. those who never consumed olive oil (HR = 0.90; 95% CI 0.82, 0.99) was inversely associated with risk for T2D	↓
							Substituting 1 tablespoon olive oil per day for margarine, butter, or mayonnaise = 5%, 8%, and 15% lower risk of T2D, respectively	↓
Guasch-Ferré et al. (2017) [21]	Spain	Investigate the associations between total fat, subtypes of dietary fat, and food sources rich in saturated FA and incidence of T2D	3349 Spanish men, 55–80 years of age and Spanish women 60–80 years of age, free from diabetes at baseline, from the PREvención con DIeta MEDiterránea (PREDIMED) study	Validated semi-quantitative FFQ	Fully adjusted model: age, sex, BMI, smoking status, educational status, leisure-time physical activity, baseline hypertension or the use of antihypertensive medication, total energy intake, alcohol intake, quartiles of fiber, protein intake, dietary cholesterol, quartiles of the other subtypes of fat, hypercholesterolemia, lipid lowering drugs, fasting plasma glucose at baseline	4.3 years	Highest quartile vs. lowest quartile of saturated and animal fat consumption (HR = 2.19: 95% CI 1.28, 3.73) were associated with risk for T2D	↑
							Total dietary fat, MUFA, PUFA, *trans* FA were not associated with T2D	↔

*BMI* body mass index, *CI* confidence interval, *DHA* docosahexaenoic acid, *EPA* eicosapentaenoic acid, *FA* fatty acid, *FFQ* food frequency questionnaire, *HR* hazard ratio, *MUFA* monounsaturated fatty acid, *OR* odds ratio, *PUFA* polyunsaturated fatty acid, *RR* relative risk, *T2D* type 2 diabetes

Implications for a connection between fish and marine *n*-3 fatty acid consumption and risk for type 2 diabetes among Westerners should be taken with caution, however, because the meta-analyses reviewed were based on many of the same heterogeneous data sets taken from various cohorts in western Europe and the USA [7–9, 10•, 11]. Whereas analyzing the data by subgroup homogenized the datasets from Asian populations, data among Westerners remained heterogeneous [10•]. No clinical trials have shown that fish consumption or marine *n*-3 fatty acids contribute to diabetes risk [5]. Further, biomarker data from prospective cohorts that aimed to assess the fatty acids present in human plasma and serum and risk for type 2 diabetes indicate no increased risk associated with marine *n*-3 fatty acids and type 2 diabetes among two Finnish cohorts and one American cohort (Table 2) [28, 29, 33]. Data from Finnish men enrolled in the Metabolic Syndrome in Men Study indicated that total *n*-3 polyunsaturated fatty acids elucidated from erythrocyte membranes were not associated with worsening hyperglycemia or type 2 diabetes [28]. Data from another cohort of Finnish men, from the Kuopio Ischaemic Heart Disease Risk Factor Study, indicated that the highest versus the lowest quartile of eicosapentaenoic acid (EPA) + docosapentaenoic acid (DPA) + docosahexaenoic acid (DHA) from serum was associated with 33% lower risk for type 2 diabetes [29]. Similarly, in a US cohort of men and women from the multi-Ethnic Study of Atherosclerosis (MESA), higher diabetes incidence was observed for individuals with serum total *n*-3 fatty acid levels below the 75th percentile [33]. No associations with diabetes were observed for those with serum total *n*-3 fatty acids above the 75% percentile [33]. Whereas these data indicate no detrimental association between serum and plasma *n*-3 fatty acids and type 2 diabetes in Western cohorts, it is still difficult to draw any definitive conclusions based on them. Data from erythrocyte membranes give short-term insight into diet, and it is unclear from the Metabolic Syndrome in Men and MESA cohorts what the dietary sources of *n*-3 fatty acids were. Current available evidence indicates that fish and marine *n*-3 fatty acid consumption may reduce the risk for type 2 diabetes in Asian populations and that more research is necessary to better understand the associations between fish, marine *n*-3 fatty acids, and type 2 diabetes among Western populations.

**Table 2:** Prospective cohort studies published between 2008 and August 2018 that investigated the association between plasma and serum fatty acids and risk for diabetes

Reference	Region	Study objective	Population	Adjustments	Time	Results	Risk
Patel et al. (2010) [22]	England	Investigate the association between FA composition and development of incident diabetes	199 cases of incident diabetes and 184 non-cases among men and women, 40–79 years of age, who resided in and around Norwich, England, from the European Prospective Investigation into Cancer and Nutrition – Norfolk study	Fully adjusted model: age, sex, BMI, family history of diabetes, physical activity, smoking status, alcohol intake	Baseline 1993–1997 Measurements taken throughout, and up until 2005	There were stronger associations with diabetes risk when FA were measured in plasma vs. erythrocytes or by FFQ. Plasma FA only are reported here	
						Third tertile vs. first tertile of total SFA (OR = 2.57; 95% CI 1.42, 4.66), palmitic acid (16:0) (OR = 2.47; 95% CI 1.37, 4.46), and Δ^9^ – SCD2 (18:1*n*-9/18:0) (OR = 2.01; 95% CI 1.12, 3.61) were associated with risk for diabetes	↑
						Third tertile vs. first tertile of stearic acid (18:0) (OR = 0.43; 95% CI 0.24, 0.79), vaccenic acid (18:1*n*-7) (OR = 0.40; 95% CI 0.22, 0.72), eicosenoic acid (20:1*n*-9) (OR = 0.48; 95% CI 0.27, 0.87), linoleic acid (18:2*n*-6) (OR = 0.50; 95% CI 0.28, 0.91), dihomo-ϒ-linolenic acid (20:3*n*-6) (OR = 0.41; 95% CI 0.23, 0.74), and Δ^5^ – desaturase (D5D) (20:4*n*-6/20:3*n*-6) (OR = 0.47; 95% CI 0.26, 0.84) were inversely associated with risk for diabetes	↓
Mozaffarian et al. (2010) [23••]	USA	Investigate whether circulating *trans*-palmitoleic acid (*trans*-16:1*n*-7) was independently related to lower metabolic risk and incident T2D	3736 men and women, 65 years of age and older, from the Cardiovascular Health Study	Age, sex, race, education, coronary heart disease, stroke, diabetes, smoking status, alcohol use, physical activity, BMI, dietary factors	14 years	*trans*-palmitoleic acid (*trans*-16:1*n*-7) was inversely associated with insulin resistance (− 16.7%, *P* < 0.001)	↓
						Fourth and highest quintiles vs. lowest quintile of *trans*-palmitoleic acid (*trans*-16:1*n*-7) (quintile 4 HR = 0.41; 95% CI 0.27, 0.64; quintile 5 HR = 0.38; 95% CI 0.24, 0.62) were inversely associated with incidence of diabetes	
Djoussé et al. (2011) [24]	USA	Investigate the association between plasma phospholipid *n*-3 FA and incident diabetes	3088 American men and women, 75 years of age on average, free from diabetes at baseline, from the Cardiovascular Health Study	Age, race, sex, clinic site, BMI, alcohol consumption, physical activity, smoking, linoleic acid, LDL-cholesterol	10.6 years	Highest quartile vs. lowest quartile of plasma α-linolenic acid (RR = 0.57; 95% CI 0.36, 0.90) was inversely associated with T2D	↓
Mozaffarian et al. (2013) [25]	USA	Investigate the associations between *trans*-palmitoleic acid (*trans*-16:1*n*-7) and metabolic risk and incident diabetes	2617 multi-ethnic men and women, 45–84 years of age, free from diabetes at baseline, from the Multi-Ethnic Study of Atherosclerosis cohort	Age, race/ethnicity, education, clinic, smoking status, alcohol use, physical activity, waist circumference, BMI	5 years	*trans*-palmitoleic acid (*trans*-16:1*n*-7) was associated with lower fasting insulin (− 9.1%, *P* = 0.002)	↓
						Highest quintile vs. lowest quintile of *trans*-palmitoleic acid (*trans*-16:1*n*-7) (HR = 0.52; 95% CI 0.32, 0.85) was inversely associated with incident diabetes	
Mahendran et al. (2013) [26]	Finland	Cross-sectional and prospective studies (prospective pool only reported here) to investigate the associations between fasting serum glycerol and FA and predictors for worsening hyperglycemia and T2D	4335 Finnish men (prospective pool only reported here), 57 years of age on average, free from diabetes at baseline from the Metabolic Syndrome in Men Study	Age, BMI, current smoking, physical activity	4.5 years	Elevated glycerol (OR = 1.18, 95% CI 1.12, 1.24), FFA (OR = 1.19, 95% CI 1.10, 1.29), MUFA (OR = 1.09, 95% CI 1.06, 1.12), SFA, and monounsaturated *n*-7 and *n*-9 (OR = 1.09, 95% CI 1.06, 1.12) predicted worsening of hyperglycemia and development of incident T2D	↑
						*n*-6 FA (OR = 0.92, 95% CI 0.89, 0.95) were associated with reduced risk for the worsening of hyperglycemia and conversion to T2D	↓
Santaren et al. (2014) [27]	USA	Prospective and cross-sectional studies (prospective pool only reported here) to investigate the associations between pentadecanoic acid (15:0) and *trans*-palmitoleic acid (*trans*-16:1*n*-7) and T2D	659 multi-ethnic men and women, 40–60 years of age, free from diabetes at baseline, from the Insulin Resistance Atherosclerosis Study	Fully adjusted model: age, sex, ethnicity, physical activity, total energy intake, total dairy intake, total hydrogenated food intake, BMI	5 years	Serum pentadecanoic acid (15:0) (OR = 0.73; 95% CI 0.56, 0.95) was inversely associated with incident diabetes risk	↓
						Serum *trans*-palmitoleic acid (*trans*-16:1*n*-7) was not associated with T2D	↔
Mahendran et al. (2014) [28]	Finland	Investigate erythrocyte membrane fatty acids as predictors of worsening hyperglycemia and incident T2D	1346 Finnish men 45–73 years of age free from diabetes at baseline from the Metabolic Syndrome in Men Study	Age, BMI, current smoking, physical activity	5 years	Palmitoleic acid (16:1*n*-7) (2.8 × 10^−7^) dihomo-ϒ-linolenic acid (20:3*n*-6) (2.3 × 10^−4^), the ratio of 16:1*n*-7 to 16:0 (as a marker of desaturase activity) (1.6 × 10^−8^), and the ratio of 20:3*n*-6 to 18:2*n*-6 (as a marker of desaturase activity) (9.4 × 10^−7^) predicted the worsening of hyperglycemia	↑
						Linoleic acid (18:2*n*-6) (*P* = 0.0015) and the ratio of 18:1*n*-7 to 16:1*n*-7 (as a marker of elongase activity) (*P* = 1.5 × 10^−9^) predicted a decrease in glucose AUC	↓
						Palmitoleic acid (16:1*n*-7) (OR = 0.54, CI 95%: 0.35, 0.82) and linoleic acid (OR = 0.54, 95% CI 0.35, 0.82) were inversely associated with T2D	↓
						*n*-3 PUFA did not show any associations with worsening hyperglycemia or T2D	↔
Virtanen et al. (2014) [29]	Finland	Investigate the associations between serum n-3 PUFA, EPA, DPA, DHA, α-linolenic acid, hair mercury and risk of incident T2D	2212 Finnish men, 42–60 years of age, free from T2D at baseline, from the Kuopio Ischaemic Heart Disease Risk Factor study	Fully adjusted model: age, examination year, BMI, family history of T2D, smoking, years of education, leisure-time physical activity, alcohol intake, serum linoleic acid	19.3 years	Highest vs. lowest quartile of EPA + DPA + DHA (HR = 0.67; 95% CI 0.51, 0.87) had inverse association with risk for T2D	↓
Lemaitre et al. (2015) [30]	USA	Investigate the association between plasma phospholipid very long-chain SFA (VLSFA) at baseline with subsequent incident diabetes	3179 men and women, 75 years of age on average, free from diabetes at baseline, from the Cardiovascular Health Study	Age, sex, race, clinic, education, smoking, alcohol use, physical activity, treated hypertension, ischemic heart disease, self-reported health status, BMI, waist circumference	18–19 years	Highest vs. lowest quartile of plasma concentration of arachidic acid (20:0) was associated with 32% lower risk for diabetes	↓
Ma et al. (2015) [31]	USA	Investigate the association of circulating palmitic acid (16:0), stearic acid (18:0), oleic acid (18:1*n*-9) and metabolic risk factors and incident diabetes	3004 men and women, 74 years of age on average, free from diabetes at baseline, from the Cardiovascular Health Study	Age, sex, race, education, clinic, smoking status, alcohol consumption, leisure-time physical activity, prevalence of ischemic heart disease, hypertension at baseline, consumption of per cent energy from protein, per cent energy from carbohydrate, total energy	18 years	Palmitic acid (16:0) (HR = 1.89. 95% CI 1.27, 2.83) and stearic acid (18:0) (HR = 1.62, 95% CI 1.08, 2.41 were associated with risk for diabetes	↑
						Oleic acid (18:1*n*-9) was not associated with risk for diabetes	↔
Lankinen et al. (2015) [32]	Finland	Investigate fasting proportions of plasma fatty acids, estimated desaturases, and elongases as predictors for worsening glycasemia and incidence of T2D	1364 Finnish men, 45–68 years of age, free from diabetes at baselines, from the Metabolic Syndrome in Men cohort	Age, BMI, smoking, physical activity at baseline, baseline fasting glucose	5.9 years	Total SFA (P = 2.3 × 10^−4^), palmitoleic acid (16:1*n*-7) (P = 2.3 × 10^−5^), dihomo-ϒ-linolenic acid (20:3*n*-6) (*P* = 1.6 × 10^−5^). estimated stearoyl-CoA desaturase 1 (*P* = 2.3 × 10^−5^), and Δ^6^-desaturase (D6D) enzyme (*P* = 9.2 × 10^−8^) activities predicted the worsening of glycaemia	↑
						PUFA, linoleic acid (18:2*n*-6) (*P* = 2.2 × 10^−4^), and elongase activity (*P* = 3.3 × 10^−8^) predicted a decrease in glucose AUC	↓
						Estimated D6D activity (HR = 1.52; 95% CI 1.21, 1.92) and dihomo-ϒ-linolenic acid (20:3*n*-6) (HR = 1.46; 95% CI 1.16, 1.84) were associated with risk of incident T2D	↑
Steffan et al. (2015) [33]	USA	Investigate the association between serum levels of non-esterified FA and risk of T2D as well as any interaction by *n*-3 FA	5697 multi-ethnic men and women, 45–84 years of age, free from diabetes at baseline, from the multi-Ethnic Study of Atherosclerosis	Fully adjusted model: age, sex, race, education, field center, current smoking, current alcohol intake, plasma *n*-3 FA, waist circumference, C-reactive protein	11.4 years	Highest quartile vs. lowest quartile of non-esterified FA (HR = 1.86; 95% CI 1.45, 2.38) was associated with incidence diabetes. Higher diabetes incidence was found across successive quartiles	↑
						Higher diabetes incidence was observed for individuals with n-3 levels below the 75th percentile	↑
						No associations were observed in those with n-3 FA ≥ 75th percentile	↔
Takkunen et al. (2016) [34]	Finland	Investigate the associations between serum fatty acid composition and T2D, insulin secretion, and insulin sensitivity	407 overweight men and women, 40–65 years of age, with impaired glucose tolerance at baseline, from the Finnish Diabetes Prevention Study	Age, sex, study group, study center, smoking, alcohol intake, waist circumference, physical activity at leisure	11 years	20:4*n*-6 (HR = 0.78; 95% CI 0.67, 0.95), 20:5*n*-3 (HR = 0.72; 95% CI 0.58, 0.88), 22:5*n*-3(HR = 0.74; 95% CI 0.69, 0.90), 22:6*n*-3 (HR = 0.73; 95% CI 0.59, 0.90), Δ^5^-desaturase (D5D) (HR = 0.78; 95% CI 0.64, 0.94), and total *n*-3 FA (HR = 0.75; 95% CI 0.57,0.86) were inversely associated with T2D	↓
Yakoob et al. (2016) [35]	USA	Investigate the associations between pentadecanoic acid (15:0), heptadecanoic acid (17:0), *trans*-palmitoleic acid *(trans*-16:1*n*-7), and incident diabetes	3333 men and women, 30–75 years of age, free from diabetes at baseline, from the Nurses’ Health Study and Health Professionals Follow-Up Study	Age, race, smoking status, physical activity, alcohol, family history of diabetes, parental history of MI, hypercholesterolemia, hypertension, menopausal status, postmenopausal hormone use, fruits, vegetables, fish, meats, whole grains, sugar-sweetened beverages, polyunsaturated fat, calcium, glycemic load, biomarker levels of *trans*-18:1, *trans*-18:2, 16:0, 18:0, BMI	15.2 years	Highest quartile vs. lowest quartile of plasma pentadecanoic acid (15:0) (HR = 0.56; 95% CI 0.39, 0.83), heptadecanoic acid (17:0) (HR = 0.57; 95% CI 0.38, 0.83), and *trans*-palmitoleic acid *(trans*-16:1*n*-7) (HR = 0.48; 95% CI 0.33, 0.70) were inversely associated with risk for diabetes	↓
Yary et al. (2016) [36]	Finland	Investigate the associations between serum *n*-6PUFA, Δ^5^-desaturase (D5D), Δ^6^-desaturase (D6D), and T2D risk	2189 men, 42–60 years of age, free from T2D at baseline, from the Kuopio Ischaemic Heart Disease Risk Factor Study	Fully adjusted model: age, examination year, family history of T2D, BMI, smoking, education, leisure-time physical activity, alcohol intake, energy, serum long chain *n*-3 PUFA concentrations	19.3 years	Highest quartile vs. lowest quartile of estimated D5D activity (HR = 0.55; 95% CI 0.41, 0.74), total *n*-6 PUFA (HR = 0.54; 95% CI 0.41, 0.73), linoleic acid (HR = 0.52; 95% CI 0.39, 0.70), and arachidonic acid (HR = 0.62; 95% CI 0.46, 0.85) were inversely associated with T2D	↓
						Higher concentrations of dihomo-ϒ-linolenic acid (20:3*n*-6) (HR = 1.38; 95% CI 1.04, 1.84) and D6D activity (HR = 1.50; 95% CI 1.14, 1.97) were associated with risk for T2D	↑
Howard et al. (2018) [37]	USA	Randomized, parallel design: decreased-fat, increased vegetable, fruit, and grain vs. comparison diet	48,835 postmenopausal women from the Women’s Health Initiative dietary intervention	8.1 years		Decreased-fat, increased vegetable, fruit, and grain group had lower rates of initiation of insulin therapy during the intervention (HR = 0.74; 95% CI 0.59, 0.94) and follow-up (HR = 0.88; 95% CI 0.78, 0.99)	↓
						In subgroup analysis of biomarkers, the intervention reduced the risk of developing glucose ≥ 100 mg/dL (OR = 0.75; 95% CI 0.61, 0.93)	

*BMI* body mass index, *CI* confidence interval, *DHA* docosahexaenoic acid, *DPA* docopentaenoic acid, *EPA* eicosapentaenoic acid, *FA* fatty acid, *FFQ* food frequency questionnaire, *HR* hazard ratio, *OR* odds ratio, *PUFA* polyunsaturated fatty acid, *RR* relative risk, *SCD* stearoul-CoA desaturase, *SFA* saturated fatty acid, *T2D* type 2 diabetes

## Associations Between Dairy Foods, Ruminant Sources of Fatty Acids, and Risk for Type 2 Diabetes

Current dietary recommendations promote dietary patterns that are low in saturated and *trans*-fat and are associated with reduced risk for chronic diseases [38]. Within those dietary patterns, dairy foods including milk, cheese, and yogurt are recommended in low-fat and fat-free varieties [38]. Whole (4% milk fat) and reduced-fat (2% milk fat) dairy foods, known collectively as “regular fat dairy,” however, and the saturated and *trans*-fatty acids that are derived from them have been associated with a reduced risk of type 2 diabetes [15, 18, 19, 23••, 35, 39].

In a prospective study of Australian men and women, whereas total dairy consumption was not associated with type 2 diabetes or metabolic syndrome, a leading risk factor for the development of type 2 diabetes, the highest versus the lowest quartile of regular-fat dairy consumption was inversely associated with metabolic syndrome [18]. Further, in a cohort of Swedish men and women, the highest versus the lowest quintile of regular-fat dairy consumption was associated with 23% less incidence of type 2 diabetes [19].

Milk fat is a complex mixture of many fatty acids of varying chain lengths with different degrees of saturation. Several of the fatty acids derived from milk have been neutrally or inversely associated with type 2 diabetes in prospective analyses. A meta-analysis designed to investigate the associations between saturated and *trans*-fat consumption and risk for chronic diseases indicated that saturated fat consumption was not associated with type 2 diabetes and ruminant derived *trans*-palmitoleic acid (*trans*-16:1, *n*-7) was associated with 42% lower risk for type 2 diabetes [39]. Four large cohorts, the Cardiovascular Health Study, the Multi-Ethnic Study of Atherosclerosis, the Nurses’ Health Study, and the Health Professionals Follow-Up Study cohorts, were included in the meta-analysis. Prospective biomarker data from these cohorts indicated inverse associations between circulating *trans*-palmitoleic acid (*trans*-16:1, *n*-7) and insulin resistance [25, 40] and incident type 2 diabetes [25, 35, 40]. Data from a smaller cohort from the USA, however, indicated that serum *trans*-palmitoleic acid (*trans*-16:1, *n*-7) had no association with risk for type 2 diabetes [27].

Prospective studies have also indicated an inverse association between saturated fats derived from dairy foods and type 2 diabetes. In Swedish men and women from the Malmö Diet and Cancer Cohort, the highest versus the lowest quintiles of saturated fatty acids with four to ten carbons, lauric acid (12:0), and myristic acid (14:0) were associated with decreased risk for type 2 diabetes [19]. The dietary assessment was an interview-based diet history method that combined a diet-recall and food-frequency questionnaire with the interview [19]. Biomarker data from plasma fatty acids has also indicated inverse associations between milk fat consumption and reduced risk for type 2 diabetes. Prospective data from 659 multi-ethnic men and women from the Insulin Resistance Atherosclerosis Study and 3333 men and women from the Nurses’ Health Study and the Health Professionals Follow-Up Study indicated that serum pentadecanoic acid (15:0), a short-term marker of milk fat consumption, was associated with 27% and 44% lower incidence of type 2 diabetes, respectively [27, 35]. Heptadecanoic acid (17:0), another short-term marker of milk fat consumption, was also inversely associated with type 2 diabetes in the Nurses’ Health Study and Health Professionals Follow-Up Study cohorts [35]. Concerns have been raised about the use of pentadecanoic acid (15:0), heptadecanoic acid (17:0), and *trans*-palmitoleic acid (*trans*-16:1, *n*-7) as biomarkers of dairy intake. These fatty acids are not exclusive to dairy fat [41•]. Further, *trans*-palmitoleic acid (*trans*-16:1, *n*-7) can be synthesized endogenously from vaccenic acid (*trans*-18:1, *n*-11), the predominant trans fatty acid isomer in dairy fats, but one also present in partially hydrogenated fats and oils [41•]. Finally, the methods used to analyze fatty acids need to be optimized to properly elucidate each fatty acid; thus, it is difficult to ensure that the biomarker data were not exaggerated by errors in methodology [41•]. Publications that used pentadecanoic acid (15:0), heptadecanoic acid (17:0), and *trans*-palmitoleic acid (*trans*-16:1, *n*-7) as biomarkers of dairy consumption did, however, correlate findings with data obtained from self-reported dietary recalls and food frequency questionnaires, which have also been brought into question. With those limitations considered, current available evidence indicates a possible inverse association between regular fat dairy consumption and risk for type 2 diabetes.

## Meat, Saturated Fat from Animal Sources, and Risk for Type 2 Diabetes

A prospective analysis of associations between food sources rich in saturated fatty acids and incidence of type 2 diabetes from 3349 Spanish men and women from the PREvención con DIeta MEDiterránea (PREDIMED) study indicated that whereas total dietary fat, monounsaturated fatty acids, polyunsaturated fatty acids, and *trans*-fatty acids were not associated with type 2 diabetes, the highest versus the lowest quartile of saturated and animal fat consumption was associated with the incidence of type 2 diabetes [21]. These findings were in agreement with those from the Malmö Diet and Cancer Cohort in which the highest versus the lowest quintile of meat consumption was associated with increased risk for type 2 diabetes [19]. Whereas these data indicate an association between meat consumption and risk for type 2 diabetes, more research is necessary to test these observations.

## Vegetable Oils, *n*-3 Monounsaturated and Polyunsaturated Fatty Acids, and Risk for Type 2 Diabetes

Prospective data from the Nurses’ Health Studies (I and II) indicated after 22 years of follow-up, that women who consumed greater than one tablespoon (approximately 8 g) of olive oil per day compared to those who never consumed olive oil had 10% lower risk of incident type 2 diabetes [20]. The predominant fatty acid in olive oil is oleic acid (18:1, *n*-9), a monounsaturated fatty acid. One prospective study of Finnish men from the Metabolic Syndrome in Men Study, however, indicated that fasting serum total monounsaturated fatty acids and oleic acid (18:1, *n*-9) predicted worsening of hyperglycemia and were associated with increased odds of developing type 2 diabetes [26]. Whereas results from these two prospective cohorts are in direct opposition to one another, as stated previously results from the PREDIMED study indicated no association between total monounsaturated fatty acid consumption and risk for type 2 diabetes [21], and results from two dietary interventions support an inverse association between monounsaturated fatty acid consumption and insulin sensitivity, a risk factor for type 2 diabetes [42, 43]. In a randomized, parallel design study conducted in 55 men and 76 women, 28 years of age on average, a moderate-fat diet containing 35–45% of energy and greater than 20% of energy as monounsaturated fatty acids decreased fasting insulin by 2.6 ± 3.6 pmol/L and the homeostasis model assessment of insulin resistance by 0.17 ± 0.13 when compared to a low-fat diet containing 20–30% of energy from fat or a control diet containing 35% of energy from fat [42]. Similarly, in a cross-over feeding intervention among 164 men and women from the Optimal Macronutrient Intake Trial to Prevent Heart Disease (OmniHeart), an unsaturated fat-rich diet made up of predominantly monounsaturated fatty acids increased the quantitative insulin sensitivity check index significantly more than a carbohydrate-rich diet similar to the Dietary Approaches to Stop Hypertension (DASH) diet or a protein-rich diet predominantly from plant sources [43].

Vegetable oils are also leading dietary sources of *n*-3 polyunsaturated fatty acids, such as α-linolenic acid, which can also be derived from nuts, milk, and meat. In a cohort of over 3000 elderly American men and women from the Cardiovascular Health Study, the highest versus the lowest quartile of plasma α-linolenic acid was associated with 43% lower risk for type 2 diabetes [24]. In a cohort of Chinese men and women from the Singapore Chinese Health Study, non-marine α-linolenic acid and *n*-3 fatty acids were inversely associated with self-reported incidence of type 2 diabetes [13]. In a cohort of over 36,000 women from the Women’s Health Study, plant-based n-3 fatty acids were not associated with incident diabetes [14]. In a cohort of Australian women from the Australian Longitudinal Study of Women’s Health, however, total *n*-3 polyunsaturated fatty acids and α-linolenic acid were associated with validated, self-reported incidence of type 2 diabetes [17]. To date, no dietary interventions have been conducted showing a detrimental effect of plant-derived *n*-3 fatty acids and type 2 diabetes. Collectively, the data indicate that vegetable oils, total monounsaturated fatty acids, and *n*-3 polyunsaturated fatty acids do not contribute to risk for type 2 diabetes.

## Conclusion: Are There Benefits to Fat Consumption on Risk for Type 2 Diabetes?

Dietary fat is a complex umbrella term that does not provide specific details regarding chain length, degree of saturation, or food source. The observational, biomarker, and clinical research published over the last decade indicates that total dietary fat consumption is not associated with risk for type 2 diabetes. Data from prospective cohort studies indicate that some fats may be particularly beneficial in reducing the risk for type 2 diabetes. In Asian populations, the consumption of fish and marine *n*-3 fatty acids has been associated with reduced risk for type 2 diabetes. In Western cohorts, reduced risk for type 2 diabetes has been associated with the consumption of regular-fat dairy foods and *trans*-palmitoleic acid (*trans-*16, *n*-7). Of the utmost importance, however, is that humans do not eat dietary fats and fatty acids in isolation. Humans eat foods, not nutrients, and based on dietary interventions that tested overall dietary patterns, low-fat dietary patterns have not been demonstrated to increase the risk for, or significantly reduce the incidence of, type 2 diabetes [37, 44]. Whereas some dietary fat may help contribute to a reduced risk for type 2 diabetes, the lifestyle recommendation to balance caloric intake with physical activity is prudent at this time.

## References

[1] Ogurtsova K, da Rocha Fernandes JD, Huang Y, Linnenkamp U, Guariguata L, Cho NH, Cavan D, Shaw JE, Makaroff LE (2017). IDF diabetes atlas: global estimates for the prevalence of diabetes for 2015 and 2040. Diabetes Res Clin Pract.

[2] Hu FB, van Dam RM, Liu S (2001). Diet and risk of type II diabetes: the role of types of fat and carbohydrate. Diabetologia.

[3] Ley SH, Hamdy O, Mohan V, Hu FB (2014). Prevention and management of type 2 diabetes: dietary components and nutritional strategies. Lancet.

[4] Knowler WC, Barrett-Connor E, Fowler SE, Hamman RF, Lachin JM, Walker EA, Nathan DM, Diabetes Prevention Program Research Group (2002). Reduction in the incidence of type 2 diabetes with lifestyle intervention or metformin. N Engl J Med.

[5] Via MA, Mechanick JI (2016). Nutrition in type 2 diabetes and the metabolic syndrome. Med Clin North Am.

[6] Acosta-Montaño Paloma, García-González Víctor (2018). Effects of Dietary Fatty Acids in Pancreatic Beta Cell Metabolism, Implications in Homeostasis. Nutrients.

[7] Muley A, Muley P, Shah M (2014). ALA, fatty fish or marine n-3 fatty acids for preventing DM?: a systematic review and meta-analysis. Curr Diabetes Rev.

[8] Wallin A, Di Giuseppe D, Orsini N, Patel PS, Forouhi NG, Wolk A (2012). Fish consumption, dietary long-chain n-3 fatty acids, and risk of type 2 diabetes: systematic review and meta-analysis of prospective studies. Diabetes Care.

[9] Wu JH, Micha R, Imamura F, Pan A, Biggs ML, Ajaz O (2012). Omega-3 fatty acids and incident type 2 diabetes: a systematic review and meta-analysis. Br J Nutr.

[10] Zheng JS, Huang T, Yang J, Fu YQ, Li D (2012). Marine N-3 polyunsaturated fatty acids are inversely associated with risk of type 2 diabetes in Asians: a systematic review and meta-analysis. PLoS One.

[11] Zhou Y, Tian C, Jia C (2012). Association of fish and n-3 fatty acid intake with the risk of type 2 diabetes: a meta-analysis of prospective studies. Br J Nutr.

[12] Kaushik M, Mozaffarian D, Spiegelman D, Manson JE, Willett WC, Hu FB (2009). Long-chain omega-3 fatty acids, fish intake, and the risk of type 2 diabetes mellitus. Am J Clin Nutr.

[13] Brostow DP, Odegaard AO, Koh WP, Duval S, Gross MD, Yuan JM, Pereira MA (2011). Omega-3 fatty acids and incident type 2 diabetes: the Singapore Chinese Health Study. Am J Clin Nutr.

[14] Djousse L, Gaziano JM, Buring JE, Lee IM (2011). Dietary omega-3 fatty acids and fish consumption and risk of type 2 diabetes. Am J Clin Nutr.

[15] Margolis KL, Wei F, de Boer IH, Howard BV, Liu S, Manson JE (2011). A diet high in low-fat dairy products lowers diabetes risk in postmenopausal women. J Nutr.

[16] Villegas R, Xiang YB, Elasy T, Li HL, Yang G, Cai H, Ye F, Gao YT, Shyr Y, Zheng W, Shu XO (2011). Fish, shellfish, and long-chain n-3 fatty acid consumption and risk of incident type 2 diabetes in middle-aged Chinese men and women. Am J Clin Nutr.

[17] Alhazmi A, Stojanovski E, McEvoy M, Garg ML (2014). Macronutrient intake and type 2 diabetes risk in middle-aged Australian women. Results from the Australian longitudinal study on Women’s health. Public Health Nutr.

[18] Louie JC, Flood VM, Rangan AM, Burlutsky G, Gill TP, Gopinath B (2013). Higher regular fat dairy consumption is associated with lower incidence of metabolic syndrome but not type 2 diabetes. Nutr Metab Cardiovasc Dis.

[19] Ericson U, Hellstrand S, Brunkwall L, Schulz CA, Sonestedt E, Wallstrom P (2015). Food sources of fat may clarify the inconsistent role of dietary fat intake for incidence of type 2 diabetes. Am J Clin Nutr.

[20] Guasch-Ferre M, Hruby A, Salas-Salvado J, Martinez-Gonzalez MA, Sun Q, Willett WC (2015). Olive oil consumption and risk of type 2 diabetes in US women. Am J Clin Nutr.

[21] Guasch-Ferre M, Becerra-Tomas N, Ruiz-Canela M, Corella D, Schroder H, Estruch R (2017). Total and subtypes of dietary fat intake and risk of type 2 diabetes mellitus in the Prevencion con Dieta Mediterranea (PREDIMED) study. Am J Clin Nutr.

[22] Patel PS, Sharp SJ, Jansen E, Luben RN, Khaw KT, Wareham NJ, Forouhi NG (2010). Fatty acids measured in plasma and erythrocyte-membrane phospholipids and derived by food-frequency questionnaire and the risk of new-onset type 2 diabetes: a pilot study in the European Prospective Investigation into Cancer and Nutrition (EPIC)-Norfolk cohort. Am J Clin Nutr.

[23] Mozaffarian D, Cao H, King IB, Lemaitre RN, Song X, Siscovick DS (2010). Trans-palmitoleic acid, metabolic risk factors, and new-onset diabetes in U.S. adults: a cohort study. Ann Intern Med.

[24] Djousse L, Biggs ML, Lemaitre RN, King IB, Song X, Ix JH (2011). Plasma omega-3 fatty acids and incident diabetes in older adults. Am J Clin Nutr.

[25] Mozaffarian D, de Oliveira Otto MC, Lemaitre RN, Fretts AM, Hotamisligil G, Tsai MY, Siscovick DS, Nettleton JA (2013). Trans-Palmitoleic acid, other dairy fat biomarkers, and incident diabetes: the multi-ethnic study of atherosclerosis (MESA). Am J Clin Nutr.

[26] Mahendran Y, Cederberg H, Vangipurapu J, Kangas AJ, Soininen P, Kuusisto J, Uusitupa M, Ala-Korpela M, Laakso M (2013). Glycerol and fatty acids in serum predict the development of hyperglycemia and type 2 diabetes in Finnish men. Diabetes Care.

[27] Santaren ID, Watkins SM, Liese AD, Wagenknecht LE, Rewers MJ, Haffner SM, Lorenzo C, Hanley AJ (2014). Serum pentadecanoic acid (15:0), a short-term marker of dairy food intake, is inversely associated with incident type 2 diabetes and its underlying disorders. Am J Clin Nutr.

[28] Mahendran Y, Agren J, Uusitupa M, Cederberg H, Vangipurapu J, Stancakova A (2014). Association of erythrocyte membrane fatty acids with changes in glycemia and risk of type 2 diabetes. Am J Clin Nutr.

[29] Virtanen JK, Mursu J, Voutilainen S, Uusitupa M, Tuomainen TP (2014). Serum omega-3 polyunsaturated fatty acids and risk of incident type 2 diabetes in men: the Kuopio ischemic heart disease risk factor study. Diabetes Care.

[30] Lemaitre RN, Fretts AM, Sitlani CM, Biggs ML, Mukamal K, King IB, Song X, Djoussé L, Siscovick DS, McKnight B, Sotoodehnia N, Kizer JR, Mozaffarian D (2015). Plasma phospholipid very-long-chain saturated fatty acids and incident diabetes in older adults: the Cardiovascular Health Study. Am J Clin Nutr.

[31] Ma W, Wu JH, Wang Q, Lemaitre RN, Mukamal KJ, Djousse L (2015). Prospective association of fatty acids in the de novo lipogenesis pathway with risk of type 2 diabetes: the Cardiovascular Health Study. Am J Clin Nutr.

[32] Lankinen MA, Stancakova A, Uusitupa M, Agren J, Pihlajamaki J, Kuusisto J (2015). Plasma fatty acids as predictors of glycaemia and type 2 diabetes. Diabetologia.

[33] Steffen BT, Steffen LM, Zhou X, Ouyang P, Weir NL, Tsai MY (2015). n-3 Fatty acids attenuate the risk of diabetes associated with elevated serum nonesterified fatty acids: the multi-ethnic study of atherosclerosis. Diabetes Care.

[34] Takkunen MJ, Schwab US, de Mello VD, Eriksson JG, Lindstrom J, Tuomilehto J (2016). Longitudinal associations of serum fatty acid composition with type 2 diabetes risk and markers of insulin secretion and sensitivity in the Finnish Diabetes Prevention Study. Eur J Nutr.

[35] Yakoob MY, Shi P, Willett WC, Rexrode KM, Campos H, Orav EJ, Hu FB, Mozaffarian D (2016). Circulating biomarkers of dairy fat and risk of incident diabetes mellitus among men and women in the United States in two large prospective cohorts. Circulation.

[36] Yary T, Voutilainen S, Tuomainen TP, Ruusunen A, Nurmi T, Virtanen JK (2016). Serum n-6 polyunsaturated fatty acids, Delta5- and Delta6-desaturase activities, and risk of incident type 2 diabetes in men: the Kuopio Ischaemic Heart Disease Risk Factor Study. Am J Clin Nutr.

[37] Howard BV, Aragaki AK, Tinker LF, Allison M, Hingle MD, Johnson KC, Manson JAE, Shadyab AH, Shikany JM, Snetselaar LG, Thomson CA, Zaslavsky O, Prentice RL (2018). A low-fat dietary pattern and diabetes: a secondary analysis from the Women’s Health Initiative dietary modification trial. Diabetes Care.

[38] United States. Department of Health and Human Services., United States. Department of Agriculture., United States. Dietary Guidelines Advisory Committee. Dietary guidelines for Americans, 2015-2020. Eighth edition. ed. Washington, D.C.: U.S. Department of Health and Human Services and U.S. Department of Agriculture; 2015.

[39] de Souza RJ, Mente A, Maroleanu A, Cozma AI, Ha V, Kishibe T, Uleryk E, Budylowski P, Schünemann H, Beyene J, Anand SS (2015). Intake of saturated and trans unsaturated fatty acids and risk of all cause mortality, cardiovascular disease, and type 2 diabetes: systematic review and meta-analysis of observational studies. BMJ.

[40] Mozaffarian D, Cao H, King IB, Lemaitre RN, Song X, Siscovick DS, Hotamisligil GS (2010). Circulating palmitoleic acid and risk of metabolic abnormalities and new-onset diabetes. Am J Clin Nutr.

[41] Ratnayake WM (2015). Concerns about the use of 15:0, 17:0, and trans-16:1n-7 as biomarkers of dairy fat intake in recent observational studies that suggest beneficial effects of dairy food on incidence of diabetes and stroke. Am J Clin Nutr.

[42] Due A, Larsen TM, Mu H, Hermansen K, Stender S, Astrup A (2008). Comparison of 3 ad libitum diets for weight-loss maintenance, risk of cardiovascular disease, and diabetes: a 6-mo randomized, controlled trial. Am J Clin Nutr.

[43] Gadgil MD, Appel LJ, Yeung E, Anderson CA, Sacks FM, Miller ER (2013). The effects of carbohydrate, unsaturated fat, and protein intake on measures of insulin sensitivity: results from the OmniHeart trial. Diabetes Care.

[44] Tinker LF, Bonds DE, Margolis KL, Manson JE, Howard BV, Larson J, Perri MG, Beresford SA, Robinson JG, Rodríguez B, Safford MM, Wenger NK, Stevens VJ, Parker LM, Women's Health Initiative (2008). Low-fat dietary pattern and risk of treated diabetes mellitus in postmenopausal women: the Women’s Health Initiative randomized controlled dietary modification trial. Arch Intern Med.

